# Referral route: a determinant of inequity for children with undiagnosed genetic diseases?

**DOI:** 10.3389/fgene.2026.1692489

**Published:** 2026-01-23

**Authors:** Zeyu Tang, Emily K. Mis, Saquib A. Lakhani

**Affiliations:** Department of Pediatrics, Pediatric Genomics Discovery Program, Yale University School of Medicine, New Haven, CT, United States

**Keywords:** disparities, genomics, race and ethnicity, rare disease, undiagnosed

## Abstract

Individuals with rare genetic diseases collectively comprise 3.5%–5.9% of the population, roughly 400 million people worldwide. Undiagnosed rare disease programs have leveraged next-generation sequencing technologies to facilitate genetic diagnoses, thereby shortening the complex diagnostic odysseys that many of these patients and their families endure. However, enrollment data suggest disparities in access to undiagnosed genetic disease programs among racial and ethnic minorities. To better understand this issue, we conducted a retrospective review of our rare undiagnosed disease program to assess whether referral route was a determinant of disparities for minoritized racial and ethnic communities. Participants enrolled in the Yale Pediatric Genomics Discovery Program from 2016 to 2022 were self-categorized into four racial and ethnic groups: Hispanic/Latinx (any race), non-Hispanic White, non-Hispanic Black/African American, non-Hispanic Other. Route of referral was classified as Inpatient, Outpatient, or Outside/Self referrals. Completion rates were the percentage of participants who completed enrollment compared to their respective group. Demographics for program participants were different from Yale-New Haven Children’s Hospital demographics, with over-representation of non-Hispanics Whites. Direct inpatient recruitment had a higher yield of Hispanic individuals, which was offset by under-representation of minoritized individuals in the Outside/Self setting. Inpatients had lower referral completion rates compared to Outpatient and Outside/Self referrals. These data suggest that the route of referral may represent different levels of access to care, and inpatient recruitment may be leveraged to promote participation by some minoritized communities. We encourage other programs to examine their cohorts for representation and identify strategies for improving participation.

## Introduction

There are roughly 7,000 known rare diseases and, collectively, patients with rare diseases comprise 3.5%–5.9% of the world’s population or roughly 400 million people (Nguengang Wakap et al., 2020; Groft et al., 2021; Baldovino et al., 2025). Each rare disease has its own pathophysiology, clinical manifestations, diagnostic testing approaches, and treatment strategies and patients with these conditions are seen by general practitioners and subspecialists alike. Although these diseases are individually rare and clinically distinct, patients frequently share many common themes in their healthcare journeys, with a major challenge being the lack of a molecular diagnosis, estimated to characterize over 50% of rare disease patients (Marwaha et al., 2022). This diagnostic uncertainty impedes appropriate management, prognostication, and family planning, and often results in tortuous diagnostic odysseys characterized by visits to multiple clinicians and by the use of assorted testing modalities in the search for a diagnosis (Ferreira, 2019; Chung et al., 2022).

The increasing availability of next-generation DNA sequencing has substantially shortened and improved the diagnostic odysseys of patients and families with rare diseases. An estimated 80% of rare diseases have an underlying genetic etiology and sequencing has led to a diagnostic yield of 25%–50% in patients with suspected rare, undiagnosed genetic conditions (Marwaha et al., 2022; Sullivan et al., 2023). This remarkable progress has been accompanied by the discovery of new diseases, reclassification of genetic variants of unknown significance, improved analytical techniques, and expanded infrastructure to conduct interval reanalysis of patients who remained undiagnosed after initial analysis (Al-Nabhani et al., 2018; Ewans et al., 2018; Jacobsen et al., 2022; Schobers et al., 2022; van Slobbe et al., 2024; Wojcik et al., 2024; Alsentzer et al., 2025; Laurie et al., 2025). However, the progress in clinical genomic diagnoses has not been equally distributed, as minoritized racial and ethnic groups are frequently under-represented in genomic programs that specialize in rare disease diagnostics (Splinter et al., 2018). Families from underserved backgrounds may face challenges such as lack of access to genetic testing, barriers to specialized care, and cultural misunderstandings, which may delay diagnosis and treatment, and result in worse overall health outcomes (Benito-Lozano et al., 2022; Kane et al., 2023; Wojcik et al., 2023; Jenkins et al., 2025). The lack of diversity is also problematic for the greater scientific community. For example, the latest version of the gnomAD database, the largest public open-access human genome reference dataset and a global resource relied upon for molecular diagnosis, is composed of 77% European ancestry; Admixed American comprises 4%, African/African American 5%, Asian 8%, and Middle Eastern ancestry 0.4% (Chen et al., 2024). One significant result of these disparities is the resulting difficulty in properly classifying variants in non-European patients with rare diseases, further delaying diagnosis and management (Popejoy and Fullerton, 2016; Popejoy et al., 2018; Sirugo et al., 2019). Although some efforts have been made to address these limitations, including diversity initiatives such as the All of Us Research Program and the Human Pangenome Project, much work remains to be done (Ramirez et al., 2022; Liao et al., 2023).

Broadly speaking, race and ethnicity, as social constructs, refer to groups of people who share certain physical characteristics or cultural heritage, respectively. They become sources of healthcare inequities for children with genetic conditions when societal biases or systemic disparities in wealth and resources limit these children’s access to early detection, appropriate treatments, and ongoing healthcare support (National Academies of Sciences and Medicine, 2023). There is a paucity of research into disparities for families with undiagnosed genetic diseases, but the limited data available suggest broad under-representation of racial and ethnic minorities (Popejoy and Fullerton, 2016; Popejoy et al., 2018; Kane et al., 2023). Specific barriers for the discrepancy in rare disease diagnostic programs are unclear, but must be understood to address inequalities.

Yale’s Pediatric Genomics Discovery Program (PGDP) combines DNA sequencing with basic science studies to find the genetic causes of undiagnosed diseases (Al-Ali et al., 2022). Most rare disease diagnostic programs recruit primarily from the outpatient setting through affiliated genetic clinics or patient self-directed enrollment (Spillmann et al., 2017). Interestingly, PGDP enrolls patients from three sources: (1) local inpatients at Yale-New Haven Children’s Hospital (YNHCH), (2) local outpatients at YNHCH subspecialty clinics, and (3) all other referral types, including a significant proportion of self-referrals. This provides a unique opportunity to test the hypothesis that referral route may impact racial and ethnic representation in rare disease programs.

## Materials and methods

We retrospectively examined PGDP participants from 1 January 2016 to 31 December 2022. Procedures for program enrollment have been previously described (Al-Ali et al., 2022). Briefly, the PGDP enrolls patients with a diverse range of phenotypes and utilizes trio exome sequencing (proband and both biological parents) combined to basic science research to support clinical genetic diagnostics and to identify previously undefined genetic conditions. Referral routes, or how the patient and family entered the program, were categorized as YNHCH Inpatients, YNHCH Outpatients (genetics and other subspecialty clinics) or Outside/Self referrals. This study included those who completed enrollment (Enrolled) and those who began the process but were lost to follow-up or otherwise deemed ineligible (Not Enrolled). Completion rates were defined as the percentage Enrolled for each referral route. Patient phenotypes were defined by review of clinical data and prior testing and participants were classified according to Human Phenotype Ontology terms (Robinson et al., 2008). Participant racial and ethnic data were obtained by self-report (for adult patients) or caregivers (for pediatric patients and those unable to provide consent and information e.g., due to intellectual disability) at the beginning of enrollment. Options reflected categories used in YNHCH electronic health record for Ethnicity: Hispanic or Latino/a/e, Not Hispanic or Latino/a/e, I Do Not Know, I Prefer Not To Share; and for Race: White, Black or African American, Asian, Middle Eastern or North African, American Indian or Native American, Native Hawaiian, Pacific Islander, I Do Not Know, I Prefer Not To Share, I Do Not See My Race Listed Here. For the purposes of this study, race and ethnicity were combined into one category, Race/Ethnicity, with four mutually exclusive groups: Hispanic/Latinx (any race), White non-Hispanic, Black/African American non-Hispanic, or Other (Marin et al., 2021). This allowed Hispanic/Latinx participants to be evaluated independently as a distinct demographic group, as we were concerned that the separate reporting of race and ethnicity may mask the true proportion of white non-Hispanic patients in comparison to other demographic groups. Non-Hispanic/Latinx participants who did not self-identify as White non-Hispanic or Black/African American non-Hispanic, as well as multi-racial patients were aggregated into Other because of the heterogeneity and small sample sizes of the individual racial and ethnic groups (Marin et al., 2021). One participant answering “N/A” was excluded; no one chose the options of “I Do Not Know,” “I Prefer Not To Share,” or “I Do Not See My Race Listed Here.”

For analysis, we used Fisher’s exact test to determine the presence of significant demographic differences for each referral route compared to the broader YNHCH patient demographics during the study period. Due to the size of the YNHCH reference cohort, a Monte Carlo simulation approach with 10,000,000 replications was used to estimate the p-values for demographic comparisons. We used Fisher’s exact test to evaluate differences in enrollment completion by (1) referral route, and (2) race and ethnicity; each sub-group was compared against the remaining overall cohort. All p-values were adjusted for multiple comparisons via Bonferroni correction for multiple comparisons. Statistical analysis was performed using R (version 4.5.0; R Foundation for Statistical Computing, Vienna, Austria) (R Core Team, 2025).

## Results

There were 242 PGDP participants and 157,041 YNHCH patients during the study period. Within PGDP, 110 identified as female (45%), the mean age at referral was 8.7 years (range from 0 (including 15 fetal referrals) to 50.4 years, with a standard deviation of 9.7 years), and 207 (86%) were under the age of 18 years at time of referral. The five most common categories of primary phenotype among the cohort (Table 1) were syndrome/multisystem disease (n = 92, 38%), abnormalities of the nervous system (n = 45, 19%), abnormalities of the cardiovascular system (n = 23, 10%), abnormalities of the digestive system (n = 14, 6%), and abnormalities of metabolism/homeostasis (11, 5%) (Supplemental 2). Phenotype information was not available for 19 (8%) of the participants with incomplete enrollment. Inpatient referrals numbered 66 (27%), Outpatient referrals were 96 (40%), and Outside/Self referrals were 80 (33%).

**TABLE 1:** Distribution of primary phenotypes of PGDP patients with undiagnosed rare diseases.

Primary phenotype	Number of patients (n)	Percentage
Syndrome/multisystem disease	92	38%
Abnormality of the nervous system	45	19%
Abnormality of the cardiovascular system	23	10%
Abnormality of the digestive system	14	6%
Abnormality of metabolism/homeostasis	11	5%
Abnormalities of the immune system	10	4%
Abnormality of the respiratory system	8	3%
Neoplasm	7	3%
Abnormality of prenatal development or birth	4	2%
Abnormality of the musculature	4	2%
Abnormality of the genitourinary system	2	1%
Abnormality of the skeletal system	2	1%
Abnormality of the connective tissue	1	<1%
No phenotype data due to incomplete enrollment	19	8%

Total percentage does not equal 100% due to rounding.

The overall PGDP demographics (Table 2) significantly differed from the general YNHCH group (p < 0.001), and analysis of referral route showed significantly different racial and ethnic demographics in Inpatient and Outside/Self routes when compared to YNHCH (p < 0.001 and p = 0.020, respectively), while Outpatient referral demographics were not significantly different (p = 0.578). Notably, Inpatient referrals included approximately twice the number of Hispanic/Latinx (n = 28, 42%) compared to the proportion expected from YNHCH demographics (23%). In contrast, there were fewer Hispanic/Latinx participants from Outpatient (n = 16, 17%) and Outside/Self groups (n = 10, 12%), where the majority were White non-Hispanic (n = 54, 56% and n = 65, 81%, respectively).

**TABLE 2:** Comparison of PGDP demographics with the YNHCH patient demographics.

Cohort	Hispanic/Latinx (any race)	White non-Hispanic	Black/African American non-Hispanic	Other non-Hispanic	Adjusted p-value (unadjusted p-value)
**PGDP (combined) (n = 242)**	**54 (22%)**	**147 (61%)**	**19 (8%)**	**22 (9%)**	<0.001* (<0.001)
Inpatient (n = 66)	28 (42%)	28 (42%)	5 (8%)	5 (8%)	0.020* (0.005)
Outpatient (n = 96)	16 (17%)	54 (56%)	11 (11%)	15 (16%)	0.578 (0.144)
Outside/self (n = 80)	10 (12%)	65 (81%)	3 (4%)	2 (3%)	<0.001* (<0.001)
**YNHCH (n = 157,071)**	**36,774 (23%)**	**78,527 (50%)**	**24,352 (16%)**	**17,418 (11%)**	-

*denotes statistical significance at p < 0.05 after Bonferroni correction for multiple comparisons.

Participants are categorized into three groups based on their referral routes: Inpatient, Outpatient, and Outside/Self referrals. Percentages are displayed for each race/ethnicity as a proportion of the referral route. The Combined, Inpatient, Outpatient, and Outside/Self cohort demographics were compared to YNHCH. Fisher’s exact test was performed with a significance level of 0.05; Monte Carlo simulation approach with 10,000,000 replications was required due to the size of the YNHCH reference cohort.

Bolded values represent results for the overall cohorts. Unbolded values represent subgroups.

A total of 193 participants (80%) completed, while 49 (20%) did not. Among the Not Enrolled participants, 23 (47%) were lost to follow-up despite outreach efforts, 15 (31%) decided to pursue alternative evaluations (clinical genetic testing, referrals to other sub-specialists), and 11 (22%) were ineligible due to the absence of required biological samples from at least one member of the exome sequencing trio (e.g., declined sample submission, biological parents were not available due to adoption or death). Enrollment completion rates (Table 3) for Inpatient were significantly lower (n = 44, 67%) than the remaining cohort (n = 176, 85%) (p = 0.01). Outpatient and Outside/Self referral routes did not have significantly different completion rates. Completion rates were not significantly different according to race and ethnicity.

**TABLE 3:** Completion rates by referral route and race and ethnicity.

Cohort	Enrolled	Not enrolled	Adjusted p-value (unadjusted p-value)
PGDP (combined) (n = 242)	80% (n = 193)	20% (n = 49)	-
By referral route			
Inpatient (n = 66)	67% (n = 44)	33% (n = 22)	0.010* (0.004)
Outpatient (n = 96)	81% (n = 78)	19% (n = 18)	1.0 (0.744)
Outside/Self (n = 80)	89% (n = 71)	11% (n = 9)	0.050 (0.017)
By race and ethnicity			
Hispanic/Latinx (any race) (n = 54)	70% (n = 38)	30% (n = 16)	0.228 (0.057)
White non-Hispanic (n = 147)	83% (n = 122)	17% (n = 25)	0.563 (0.1408)
Black non-Hispanic (n = 19)	74% (n = 14)	26% (n = 5)	1.0 (0.551)
Other non-Hispanic (n = 22)	86% (n = 19)	14% (n = 3)	1.0 (0.581)

*denotes statistical significance at p < 0.05 after Bonferroni correction for multiple comparisons.

Participants are sub-grouped by (1) referral route, and (2) race and ethnicity. The percentages of completed enrollments are listed for each sub-group. Fisher’s exact test was used for each sub-group with a significance level of 0.05.

Bolded values represent results for the overall cohorts. Unbolded values represent subgroups.

## Discussion

Disparities in access to rare disease diagnostic programs are well-documented, prolonging diagnostic odysseys, perpetuating inequalities, and limiting scientific advancements (Popejoy and Fullerton, 2016; Popejoy et al., 2018; Walley et al., 2018; Fatumo et al., 2022). Providers across specialties have struggled with understanding the contributing factors in order to address this significant problem (Jenkins et al., 2025). Although prior studies have explored other barriers to diversity, our study leveraged the different recruitment strategies available for the Yale PGDP to examine whether referral route was a potential factor in recruitment diversity. We found that Inpatient recruitment contributed significantly to ethnic and racial diversity of our rare disease program, gains that were offset by lower diversity from Outside/Self referrals. These findings corroborate previous work on the subject that indicate a concerning disparity in access to advance genetic diagnostic care, while providing a perspective that may offer a potential solution (Walley et al., 2018; Kane et al., 2023).

The identification of recruitment route as an important determinant for the recruitment of racial and ethnic minorities may reflect their greater inpatient utilization for chronic illnesses or a decreased outpatient access in minoritized populations or a combination of the two (Slain et al., 2024). Racial and ethnic minorities also have delayed presentation to genetics clinics, and present with more severe phenotypes relative to white non-Hispanic patients (Wojcik et al., 2023). In some undiagnosed disease programs, the ambulatory referral base makes the bulk of enrollments and some require letters of support and personal narratives for enrollment (Spillmann et al., 2017). Our study found that the outpatient referral method of recruitment had a mixed outcome: ambulatory referrals within our health system were demographically proportionate, while self-referrals and referrals from those outside our health system had the lowest racial and ethnic diversity. Indeed, our anecdotal experience with the Outside/Self cohort is that these participants are frequently referred to as “motivated,” typically indicating some combination of time, wealth, contacts, or savvy navigating the medical system, characteristics less often found in underrepresented populations (Walley et al., 2018; Jackson et al., 2019; Palleiko et al., 2020).

Interestingly, inpatient referrals to the Yale PGDP had the greatest diversity, suggesting that an emphasis on inpatient recruitment may improve racial and ethnic diversity for rare disease diagnostic programs. Multiple prior studies have already supported the use of inpatient genomic testing for clinical diagnoses in children with undiagnosed genetic conditions (Gubbels et al., 2020; Schroeder et al., 2021; Bowling et al., 2022). The contrast between inpatient and outpatient recruitment may be due to clustered access to multidisciplinary teams, closer monitoring by healthcare staff, and expedited testing relative to ambulatory care. Although these data suggest that inpatient settings may offer more inclusive recruitment for rare and undiagnosed disease programs, we also identified lower enrollment completion rates for inpatients. The reasons remain unclear but may include families focusing their attention on illness, insufficient resources to subsequently follow up, language barriers, gaps in health literacy, or inadequate outreach efforts by our program. Future research is needed to address these completion disparities.

To our knowledge, this is the first study to identify discrepancies in referral demographics and describe demographic data by recruitment source in patients with undiagnosed genetic diseases. These findings underscore the need to analyze referral pathways to address under-representation and promote equity. We encourage other rare disease programs to systematically analyze their referral networks for underrepresentation of minoritized communities, which includes examining recruitment routes and implementing targeted outreach strategies to ensure equitable participation.

## Data Availability

Deidentified raw data supporting the conclusions of this article will be made available by the authors, without undue reservation.
